# A recurrent Gaussian quantum network for online processing of quantum time series

**DOI:** 10.1038/s41598-024-61004-7

**Published:** 2024-05-29

**Authors:** Robbe De Prins, Guy Van der Sande, Peter Bienstman

**Affiliations:** 1 Photonics Research Group, Ghent University - imec, Technologiepark-Zwijnaarde 126, 9052 Gent, Belgium; 2 Applied Physics Research Group, Vrije Universiteit Brussel, Pleinlaan 2, 1050 Brussels, Belgium

**Keywords:** Optical physics, Quantum physics

## Abstract

Over the last decade, researchers have studied the interplay between quantum computing and classical machine learning algorithms. However, measurements often disturb or destroy quantum states, requiring multiple repetitions of data processing to estimate observable values. In particular, this prevents online (real-time, single-shot) processing of *temporal* data as measurements are commonly performed during intermediate stages. Recently, it was proposed to sidestep this issue by focusing on tasks with quantum output, eliminating the need for detectors. Inspired by reservoir computers, a model was proposed where only a subset of the internal parameters are trained while keeping the others fixed at random values. Here, we also process quantum time series, but we do so using a Recurrent Gaussian Quantum Network (RGQN) of which *all* internal interactions can be trained. As expected, this increased flexibility yields higher performance in benchmark tasks. Building on this, we show that the RGQN can tackle two quantum communication tasks, while also removing some hardware restrictions of the currently available methods. First, our approach is more resource efficient to enhance the transmission rate of quantum channels that experience certain memory effects. Second, it can counteract similar memory effects if they are unwanted, a task that could previously only be solved when redundantly encoded input signals could be provided. Finally, we run a small-scale version of the last task on Xanadu’s photonic processor Borealis.

## Introduction

In the pursuit of improved data processing, there is an increasing emphasis on combining machine learning (ML) techniques with quantum computing (QC). Building on the established belief that quantum systems can outperform classical ways of computing^1^, quantum machine learning (QML)^2^ provides a methodology for identifying applications for quantum computers where the target algorithm is learned (i.e. trained) rather than designed.

In classical machine learning, algorithms such as recurrent neural networks (RNNs)^3,4^, transformers^5,6^, long short-term memory (LSTM) networks^7^, and reservoir computing (RC)^8^ have led to state-of-the-art performances in natural language processing, computer vision, and audio processing. This makes them good sources of inspiration for new QML models.

However, the common use of projective measurements in quantum computing leads to the requirement of processing the same input data multiple times to estimate the expectation values of detected observables. It is known that this can lead to an exponential decrease in achievable computational complexity^9^, and this poses even more of a fundamental bottleneck for *temporal* models. As the detections are often carried out at intermediate processing stages, this leads to back-actions on the state of the quantum system. On the one hand, this gives rise to laborious operating procedures and large overheads^10^. On the other hand, it prevents one from performing online time series processing (i.e. constantly generating output signals in real-time, based on a continuous stream of input signals). This restricts the use of temporal models in fields like quantum communication, where online operation is of great importance.

Recently, an approach was introduced that proposes to sidestep this detection issue by performing online processing of quantum states and thereby removing the need for detectors^11^. The model was inspired by the concept of RC^8^, where random dynamical systems, also called reservoirs, are made to process temporal input data. RC research has demonstrated that training only a simple output layer to process the reservoir’s output signals can achieve state-of-the-art performance in various computational tasks while significantly reducing training costs. Building on this idea, Ref.^11^ tackled several computational tasks using a random network of harmonic oscillators and training only the interactions between that network and some input-carrying oscillators.

Here, we introduce a Recurrent Gaussian Quantum Network (RGQN). Inspired by RNNs rather than RC, we choose to train all interactions within the RGQN rather than keeping a subset of these interactions fixed. Specifically, we train all interactions within the Gaussian state formalism^12^, thereby excluding non-Gaussian components like the Kerr gate and the cubic phase gate. This last restriction is beneficial as it allows us to use simple numerical tools while, on the experimental side, the RGQN can be implemented using optical components that are readily available in the laboratory^13–15^. We first compare our model with the findings of Ref.^11^ by conducting numerical simulations of two computational tasks: the short-term quantum memory (STQM) task and the entangler task. We will provide detailed definitions of these tasks in the following sections. They respectively serve as benchmarks to assess the RGQN’s linear memory capabilities and its ability to entangle different states in a time series. As expected, we will show that the RGQN outperforms the RC-inspired approach at these benchmark tasks.

More interestingly, we demonstrate that the increased flexibility of our model makes it well-suited to tackle two different tasks within the domain of quantum communication, where optics naturally is the leading hardware platform. We will show that the RGQN can remove some of the hardware restrictions of the currently available methods for these tasks. First, we show that the RGQN can enhance the capacity of a quantum memory channel. In such a channel, subsequent signal states are correlated through interactions with the channel’s environment. Our network achieves this enhancement by generating an entangled quantum information carrier. Indeed, it is known that the asymptotic transmission rate of memory channels can be higher than the maximal rate achieved by separable channel uses. It is said that the capacity of such channels is ‘superadditive’. For a bosonic memory channel with additive Gauss-Markov noise^16^, it was previously shown that the generation of such entangled carriers can be performed sequentially (i.e. without creating all channel entries all at once) while achieving near-optimal enhancement of the capacity^17^. Our model achieves the same result while having a simpler encoding scheme (e.g., without Bell measurements) and being more versatile, as it is known reconfigurable circuits can adapt to fabrication imperfections^18,19^.

Moreover, we show that a RGQN can also compensate for unwanted memory effects in quantum channels (the so-called quantum channel equalization or QCE task). Existing work on this task required the availability of redundantly encoded input signals^11^. This undermines the practicality of the method. Moreover, such a redundant encoding is impossible without full prior knowledge of the input states (e.g. when they result from a previous quantum experiment that is not exactly repeatable) because quantum states cannot be cloned. Here, we show that the increased flexibility of the RGQN allows us to lift the restriction of redundant encoding. Additionally, we find that the RGQN’s performance can be improved by allowing the reconstruction of the channel’s input to be performed with some delay.

As the RGQN does not contain any non-Gaussian components, it can be constructed using optical components that are readily available in the laboratory^13–15^. To confirm this, we conducted a small-scale version of the QCE task on the recently introduced photonic processor Borealis^15^. However, our results are constrained by the limited tunability of Borealis’ phase modulators.

The rest of this paper is structured as follows. We introduce our RGQN model in the first section and benchmark it with the STQM and entangler tasks in the second section. The third section shows that the RGQN achieves superadditivity in a bosonic memory channel and that it tackles the QCE task without redundantly encoded input signals. Finally, we present the experimental QCE results.

## Model

**Figure 1:**
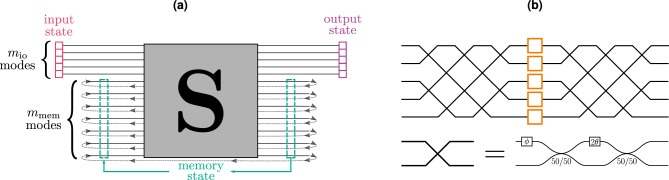
RGQN model. (**a**) Quantum states are repeatedly sent in the upper $m_\text {io}$ modes. These input modes are combined with $m_\text {mem}$ memory modes and sent through a symplectic circuit ${\textbf{S}}$. Afterwards, the state on the upper $m_\text {io}$ modes is collected as output, while the remaining $m_\text {mem}$ modes are looped back to the left side of ${\textbf{S}}$. (**b**) The symplectic circuit ${\textbf{S}}$ can be constructed as a linear interferometer, a set of single-mode squeezers (orange boxes), and a second linear interferometer^20^.

Our RGQN model is presented in Fig. 1a. It incorporates a generic *m*-mode circuit ${\textbf{S}}$ that consists of beam splitters, phase shifters, and optical squeezers. Such a circuit can be described by a symplectic matrix. Hence, we will further refer to it as a symplectic circuit. As shown in Fig. 1b, any symplectic circuit can be constructed as a linear interferometer, followed by a set of single-mode squeezers and a second linear interferometer. This is known as the Bloch-Messiah decomposition^20^. The two linear interferometers can be further decomposed into a set of beamsplitters and phase shifters using a rectangular decomposition^21^.

The $m_\text {io}$ upper modes at the left (right) side of ${\textbf{S}}$ are the input (output) modes of the RGQN. The remaining modes of ${\textbf{S}}$ are connected from left to right using $m_\text {mem}= m - m_\text {io}$ delay lines. The delay lines are equally long and we will further denote them as ‘memory modes’. To perform a temporal task, we send a time series of quantum states (e.g., obtained from encoding classical information or from a previous quantum experiment) to the input modes of the RGQN. The temporal spacing between the states is chosen equal to the propagation time of ${\textbf{S}}$ and the delay lines, such that we can describe the RGQN operation in discrete time. Because of the memory modes, output states depend on multiple past input states, which grants the RGQN some memory capacity. By training the circuit ${\textbf{S}}$ (essentially training the parameters of its constituent gates), the RGQN can learn to process temporal data.

In further sections, we sometimes restrict ${\textbf{S}}$ to be *orthogonal* symplectic. Such a circuit only comprises beam splitters and phase shifters, excluding optical squeezers. When applicable, we will denote the circuit as ${\textbf{O}}$.

Note that, in contrast to quantum RNN models like Ref.^22^, no detectors are present in our model. Although we will need to use detectors to train the hardware setup, they will be left out at inference time. As discussed before, this allows for *online* operation, which means our model focuses on tackling a conceptually different set of tasks than currently existing quantum temporal models.

## Benchmark tasks

### Short-term quantum memory task

**Figure 2:**
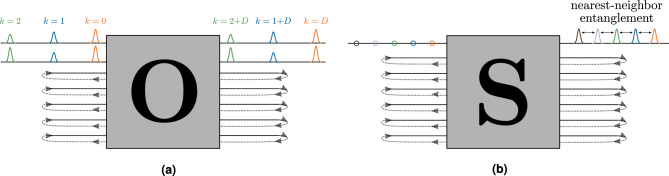
Setups for the benchmark tasks. (**a**) STQM task with *m*_io_ = 2. The RGQN consists of an orthogonal symplectic network **O**. Pulses of different colors represent a time series of quantum states. A state that is sent into the RGQN at iteration k should appear at the output at iteration *k* + *D*. (**b**) Entangler task for *m*_io_ = 1 and spacing *S* = 1. Circles of different colors represent an input time series of vacuum states. Pulses of different colors are entangled output states.

The goal of the short-term quantum memory (STQM) task is to recall states that were previously fed to the RGQN after a specific number of iterations, denoted by $D$. This task is visualized in Fig. 2a for the case where $m_\text {io}=2$ and the RGQN consists of an orthogonal circuit. Note that if we were to use a general symplectic network instead of an orthogonal one, optical squeezers could be added and optimized, such that the results would be at least equally good. However, we will show that we can reach improved performance without including optical squeezers in the RGQN, which is beneficial for an experimental setup.

**Figure 3:**
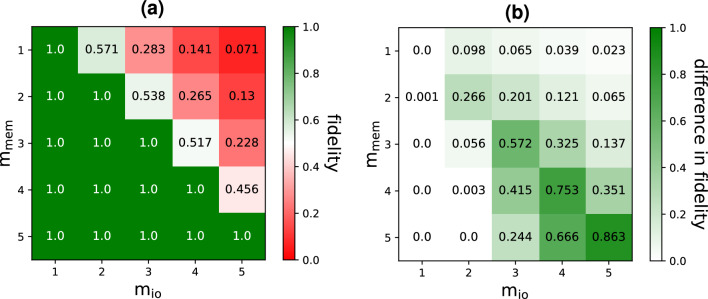
STQM performance for $D=1$ and for different values of $m_\text {io}$ and $m_\text {mem}$. (**a**) Shows the average fidelity between a desired output state and a state resulting from the RGQN. In (**b**), the corresponding results achieved in Ref.^11^ are subtracted from our results.

We focus our attention on the case where $D=1$. The input states are chosen randomly from a set of squeezed thermal states (more details in Methods). Fig. 3a shows the average fidelity^23^ between an input state at iteration *k* and an output state at iteration $k+D$, as a function of $m_\text {mem}$ and $m_\text {io}$. We see that the RGQN perfectly solves the STQM task if $m_\text {io}\le m_\text {mem}$. This is easy to understand as ${\textbf{O}}$ can be trained to perform several SWAP operations (i.e. operations that interchange the states on two different modes). More specifically, the RGQN can learn to swap every input mode with a different memory mode, such that the input state is memorized for a single iteration before being swapped back to the corresponding output mode. For $m_\text {io}> m_\text {mem}$, such a SWAP-based circuit is not possible, leading to less than optimal behavior of the RGQN.

In Fig. 3b, the fidelity values obtained by the RC-inspired model of Ref.^11^ are subtracted from our results. Across all values of $m_\text {mem}$ and $m_\text {io}$, we observe that the RGQN scores equally well or better. Although Ref.^11^ also achieves a fidelity of 1 for certain combinations of $m_\text {io}$ and $m_\text {mem}$, the set of these combinations is smaller than for the RGQN. Moreover, it is important to note that the RC-inspired design limits the number of trainable parameters, making a SWAP-based solution impossible in general. As a result, prior to training, it is more challenging to guarantee optimal performance of the RC-inspired model, while this is not the case for the RGQN.

### Entangler task

The objective of the entangler task is to entangle different states of a time series that were initially uncorrelated. The performance of this task is evaluated based on the average logarithmic negativity between output states at iterations *k* and $k+S$. Negativity^12^ is an entanglement measure for which higher values indicate greater levels of entanglement between the states. Note that if we consider output states with spacing $S=1$, then we aim to entangle nearest-neighbor states. This last task is visualized in Fig. 2b for the case where $m_\text {io}=1$. We choose vacuum states as input and hence the circuit ${\textbf{S}}$ should *not* be orthogonal as we want to generate states with nonzero average photon numbers.

**Figure 4:**
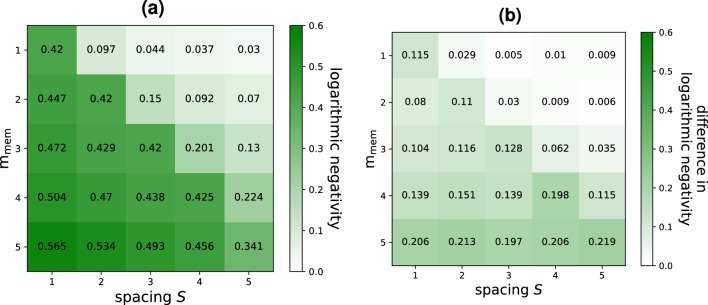
Entangler task performance for $m_\text {io}=1$ and for different values of *S* and $m_\text {mem}$. (**a**) Shows the logarithmic negativity resulting from the RGQN. In (**b**), the corresponding results achieved in Ref.^11^ are subtracted from our results.

For $m_\text {io}=1$, Fig. 4a displays the average logarithmic negativity obtained by the RGQN for various values of $m_\text {mem}$ and *S*. For a given spacing, the performance increases with $m_\text {mem}$. This can be attributed to the fact that a higher value of $m_\text {mem}$ leads to a bigger circuit ${\textbf{S}}$, such that more entangling operations can be applied. It can also be seen that the performance roughly stays the same along the diagonal ($S=m_\text {mem}$) and along lines parallel to the diagonal. This can be explained by the findings of Fig. 3, which indicate that increasing $m_\text {mem}$ can effectively address the increased linear memory requirements of the task that arise from increasing *S*. Finally, our comparison with the RC-inspired model proposed in Ref.^11^, as shown in Fig. 4b, indicates that the RGQN performs better, owing to its larger number of trainable parameters. For instance, when $m_\text {mem}=5$ and $S=1$ the RGQN achieves a logarithmic negativity of 0.565 whereas the RC-inspired model reaches a value of 0.359. These values correspond to those of two-mode squeezed vacuum states with squeezing amplitudes 0.283 and 0.180, respectively.

## Quantum communication tasks

Whereas the last section confirmed that the performance of the benchmark task can be increased by using the RGQN instead of the RC-inspired strategy, in this section we show that the higher flexibility of our model makes it well-suited to tackle quantum communication tasks. For two such tasks, the RGQN will remove some hardware restrictions of the currently available methods.

### Superadditivity

First, we show that the RGQN can enhance the transmission rate of a quantum channel that exhibits memory effects. When a state is transmitted through such a ‘memory channel’, it interacts with the channel’s environment. As subsequent input states also interact with the environment, correlations arise between different channel uses. Contrary to memoryless channels, it is known that the transmission rate of memory channels can be enlarged by providing them with input states that are entangled over subsequent channel uses^24^, a phenomenon that is known as ‘superadditivity’. Here, we aim to create such entangled input states using our RGQN.

Note the similarity with the definition of the entangler task. Now however, the goal is not to create maximal entanglement between the different states, but rather a specific type of entanglement that depends on the memory effects of the channel and that will increase the transmission rate.

**Figure 5:**
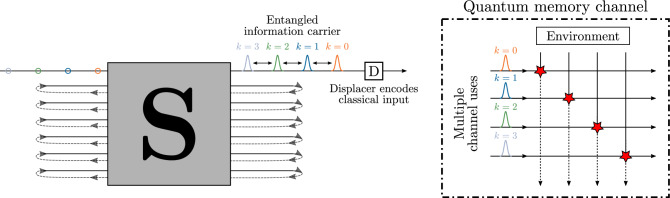
Setup for the superadditivity task. A RGQN (with $m_\text {io}=1$) transforms vacuum states into a quantum information carrier that is entangled across different time bins. A displacer (D) modulates this carrier to encode classical input information. The resulting signal is sent to a bosonic memory channel^16^. A number of *K* consecutive uses of the channel are modeled as a single parallel *K*-mode channel. The channel’s environment introduces noise  that leads to correlations between the successive channel uses. As a result of the entangled carrier, the transmission rate of the channel can be enhanced.

The setup for the ‘superadditivity task’ is shown in Fig. 5. A RGQN with $m_\text {io}= 1$ transforms vacuum states into an entangled quantum time series. Information is encoded by displacing each individual state of the series over a continuous distance in phase space. These distances are provided by a classical complex-valued information stream. Their probabilities follow a Gaussian distribution with zero mean and covariance matrix $\varvec{\gamma }_{\textrm{mod}}$.

The resulting time series is sent through a memory channel. A number of *K* consecutive uses of the channel are modeled as a single parallel *K*-mode channel. The memory effects we consider here are modeled by correlated noise emerging from a Gauss-Markov process^16^. The environment has the following classical noise covariance matrix $\varvec{\gamma }_{\textrm{env}}$:

1 $$\begin{aligned}{} & {} \varvec{\gamma }_{\textrm{env}} = \left( \begin{array}{cc}{\textbf{M}}(\phi ) &{} 0 \\ 0 &{} {\textbf{M}}(-\phi )\end{array}\right) ~, \end{aligned}$$

2 $$\begin{aligned}{} & {} M_{i j}(\phi ) = N \phi ^{|i-j|} ~. \end{aligned}$$

Here, $\phi \in [0, 1)$ denotes the strength of the nearest-neighbor correlations and $N \in {\mathbb {R}}$ is the variance of the noise. In Eq. (1), ${\textbf{M}}(\phi )$ correlates the *q* quadratures, while ${\textbf{M}}(-\phi )$ anti-correlates the *p* quadratures.

The transmission rate of the channel is calculated from the von Neumann entropy of the states that pass through the channel (i.e. from the Holevo information). Here we adopt the approach and the parameter values outlined in Ref.^16^.

Note that the average photon number that is transmitted per channel use (${\bar{n}}$) has a contribution from both the RGQN (i.e. from its squeezers) and from the displacer. Given a value for ${\bar{n}}$, the transmission rate is maximized by training both the circuit ${\textbf{S}}$ and $\varvec{\gamma }_{\textrm{mod}}$ under the energy constraint imposed by ${\bar{n}}$. Nonzero squeezing values are obtained, leading to an information carrier. This highlights the counter-intuitive quantum nature of the superadditivity phenomenon: by spending a part of the available energy on the carrier generation rather than on classical modulation, one can reach higher transmission rates, something that has no classical analog.

**Figure 6:**
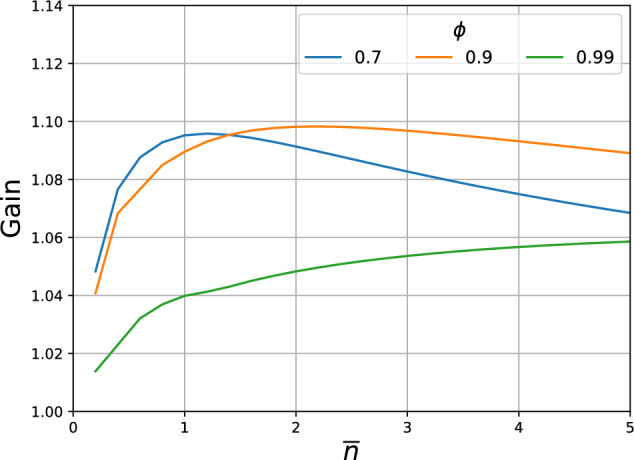
Performance of the superadditivity task for 30 channel uses. The gain in transmission rate is plotted as a function of the average photon number per use of the channel (${\bar{n}}$) and for different values of the noise correlation parameter ($\phi$). Additional parameters are chosen as follows: $m_\text {io}=m_\text {mem}=1$, $N={\bar{n}}/3$.

We now define a quality measure for the superadditivity task. The gain *G* is the ratio of the achieved transmission rate to the optimal transmission rate for separable input states. For 30 channel uses, Fig. 6 shows *G* as a function of the average photon number ${\bar{n}}$ per use of the channel and for different values of the correlation parameter $\phi$. We take the signal-to-noise ratio ${\text{SNR}} = {\bar{n}}/N = 3$, where *N* is defined in Eq. (2). We observe that superadditivity is achieved, as the gain is higher than 1 and can reach as high as 1.10. These results agree with the optimal gain values that were derived in prior numerical studies of this memory channel (cfr. Fig. 7 of Ref.^25^).

While a scheme already exists to generate carriers sequentially^17^ (i.e., generating carriers without creating all channel entries simultaneously), our model provides a simpler and more versatile alternative. Unlike the existing scheme, our model eliminates the need for Bell measurements, while achieving the same near-optimal gains. Additionally, it is known that reconfigurable circuits can learn to compensate for fabrication imperfections as these imperfections are taken into account during training^18,19^.

### Quantum channel equalization

In this section, we use the RGQN as a model for a quantum memory channel. This time, we assume its memory effects to be unwanted (unlike the previous section) and compensate for them by sending the channel’s output through a second RGQN instance.

**Figure 7:**
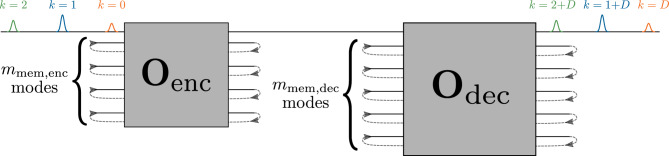
Setup for the QCE task when $m_\text {io}=1$ and for a delay $D$. Pulses of different colors represent a time series of quantum states. The encoder and decoder respectively consist of orthogonal symplectic networks ${\textbf{O}}_\text {enc}$ and ${\textbf{O}}_\text {dec}$. ${\textbf{O}}_\text {enc}$ is initialized randomly and kept fixed. ${\textbf{O}}_\text {dec}$ is trained such that an input state that is sent into the encoder at iteration *k* appears at the output of the decoder at iteration $k+D$.

Figure 7 shows the setup for the quantum channel equalization (QCE) task in more detail. An ‘encoder’ RGQN acts as a model for a memory channel. Because such channels normally do not increase the average photon number of transmitted states, we restrict the encoder’s symplectic circuit to be orthogonal and denote it as ${\textbf{O}}_\text {enc}$. This circuit is initialized randomly and will not be trained later. A second ‘decoder’ RGQN is trained to invert the transformation caused by the encoder. Similar to the STQM task, we will show that an orthogonal symplectic circuit ${\textbf{O}}_\text {dec}$ is enough to lead to the desired performance, without requiring optical squeezers, which is beneficial for experimental realizations. We will further denote the number of memory modes of the encoder and decoder as $m_\text {mem,enc}$ and $m_\text {mem,dec}$ respectively. Finally, we introduce a delay of $D$ iterations between the input and output time series, similar to the definition of the STQM task (see Fig. 2a).

Assume for a moment that the input time series of the encoder only consists of a single state, i.e. we are looking at an impulse response of the system. We send this state to the encoder at iteration 0, and expect the decoder to reconstruct it at iteration $D$. However, a part of the input state may be initially stored in the encoder’s memory modes. By choosing $D>0$, multiple states are sent from the encoder to the decoder, thereby depleting the encoder’s memory modes. When we increase $D$, more information about the original input state reaches the decoder by the time it needs to start the reconstruction process. A similar reasoning applies when the input time series consists of multiple states. This approach effectively addresses the challenge posed by the no-cloning principle, which prevents the decoder from accessing information stored in the encoder’s memory or in the correlations between the encoder’s memory and output.

For the RC-inspired model of Ref.^11^, only the case where $D=0$ was considered. The no-cloning problem was addressed by redundantly encoding the input signals of the encoder. I.e., multiple copies of the same state were generated based on *classical* input information and subsequently fed to the model through different modes (‘spatial multiplexing’) or at subsequent iterations (‘temporal multiplexing’). Here, we show that taking $D>0$ allows us to solve the QCE task without such redundancy, ultimately using each input state only once. This not only simplifies the operation procedure but also enables us to perform the QCE task without prior knowledge of the input states, which is often missing in real-world scenarios such as quantum key distribution. As these input states cannot be cloned, our approach significantly increases the practical use of the QCE task. It is important to note that such an approach where $D>0$ was also attempted for the RC-inspired model^26^, but this was unsuccessful, which can be attributed to its limited number of trainable interactions.

Additionally, we will show that the QCE performance of the RGQN is influenced by two key factors: the memory capacity of the decoder (as determined by the value of $m_\text {mem,dec}$), and the response of the encoder to a single input state (observed at the encoder’s output modes).

More formally, we measure the impulse response of the encoder by sending in a single squeezed vacuum state (with an average photon number of ${\bar{n}}_\text {impulse}$) and subsequently tracking the average photon number $h_{\text {enc}}$ in its output modes over time. We denote the impulse response at iteration *k* by $h_{\text {enc}}^k$.

We now define:

3 $$\begin{aligned} I_{\text {enc}} = \frac{1}{{\bar{n}}_\text {impulse}} \sum _{k=0}^{D} h_{\text {enc}}^k ~. \end{aligned}$$

$I_{\text {enc}}$ is a re-normalized cumulative sum that represents the fraction of ${\bar{n}}_\text {impulse}$ that leaves the encoder before the decoder has to reconstruct the original input state.

**Figure 8:**
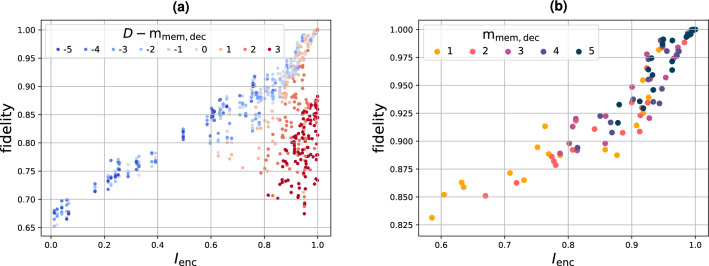
QCE performance for 20 randomly initialized encoders that consist of 2 memory modes. The results are shown as a function of $I_{\text {enc}}$, i.e. the fraction of the photon number of a certain input state that reaches the decoder within $D$ iterations. In (**a**), we consider $D\in \{0, 1, ...,m_\text {mem,dec}+m_\text {mem,enc}+1\}$ and $m_\text {mem,dec}\in \{1,2,...,5\}$. In (**b**), the optimal value of $D$ is chosen (given an encoder and $m_\text {mem,dec}$).

We now consider 20 randomly initialized encoders with $m_\text {mem,enc}=2$. The input states are randomly sampled from a set of squeezed thermal states (more details in Methods). Figure 8a shows the average fidelity^23^ between an input state of the encoder at iteration *k* and an output state of the decoder at iteration $k+D$ as a function of $I_{\text {enc}}$ and for different values of $D- m_\text {mem,dec}$. We see that if $D\le m_\text {mem,dec}$ (blueish dots), the decoder potentially has enough memory, and the quality of constructing the input states increases as the decoder receives more information from the encoder (i.e. as $I_{\text {enc}}$ increases). If $D> m_\text {mem,dec}$ (reddish dots), we ask the decoder to wait for a longer time before starting to reconstruct the input. This explains why the dots are clustered on the right side of the diagram because more information about the input will be received and $I_{\text {enc}}$ will be higher. On the other hand, if the delay is too long, it will exceed the memory of the decoder, and the input will start to be forgotten. This explains that the darkest dots with the longest delay have the worst performance.

Note that $D$ is a hyperparameter that can be chosen freely. Also note that the optimal choice for the value of $D$ is not necessarily $D= m_\text {mem,dec}$ (light grey dots) and the actual optimum depends on the exact configuration of the encoder. Figure 8b shows a subset of the results in Fig. 8a, where the optimal value of $D$ is chosen for every encoder initialization and every value of $m_\text {mem,dec}$. We conclude that the task can be tackled without redundantly encoded input signals. As discussed earlier, such redundancy was required in Ref.^11^, but often cannot be provided in real-world scenarios. We further observe that the performance increases with both $m_\text {mem,dec}$ and $I_{\text {enc}}$. For $m_\text {mem,dec}=3$, all 20 encoders are equalized better than is done in Ref.^11^.

### Experimental demonstration of quantum channel equalization

**Figure 9:**
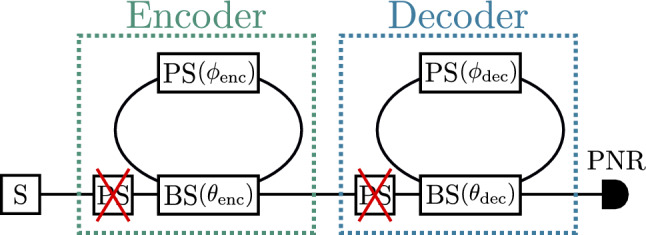
Setup for the QCE task where both the encoder and decoder have a single memory mode. The matrices ${\textbf{O}}_\text {enc}$ and ${\textbf{O}}_\text {dec}$ are decomposed using beam splitters (BS) and phase shifters (PS). A squeezer (S) is used to generate input states, while the output states are measured using a photon-number-resolving (PNR) detector. As the PSs outside of the loops do not influence the PNR results, they can be disregarded.

As the RGQN does not contain any non-Gaussian components, it can be constructed using optical components that are readily available in the laboratory^13–15^. In this section, we perform the QCE task on the recently introduced quantum processor Borealis^15^. Because of hardware restrictions, we only consider the case where $m_\text {mem,enc}= m_\text {mem,dec}= 1$. The setup for this task is visualized in Fig. 9. The input time series consists of squeezed vacuum states (whereas squeezed *thermal* states were previously used to simulate the QCE task). Both the encoder and decoder are decomposed using beam splitters and phase shifters. Here we use the following definitions for those respective components:

4 $$\begin{aligned} BS(\theta )= & {} e^{\theta (a_i a_j^\dagger - a_i^\dagger a_j)}~, \end{aligned}$$

5 $$\begin{aligned} PS(\phi )= & {} e^{i \phi a_i^\dagger a_i}~, \end{aligned}$$

where $a_i^\dagger$ ($a_i$) is the creation (annihilation) operator on mode *i*. Note that the transmission amplitude of the beamsplitter is $\cos (\theta )$.

Whereas Fig. 8 presented the results of training the decoder, here we will visualize a larger part of the cost function landscape (including sub-optimal decoder configurations). By doing so, we are agnostic to the exact training procedure, such as the parameter-shift rule^27,28^. Note that while evaluating a certain point of the cost function landscape, i.e. while processing a single time series, the parameters of the beam splitters and phase shifters are kept fixed. Hence, in Fig. 9, the measurement results are not influenced by the phase shifters outside of the loops (i.e. outside of the memory modes). These components can be disregarded. Consequently, we can parameterize the encoder (decoder) using only a beam splitter angle $\theta _{\text {enc}}$ ($\theta _{\text {dec}}$) and a phase shift angle $\phi _{\text {enc}}$ ($\phi _{\text {dec}}$).

We use a photon-number-resolving (PNR) detector to detect output states. This provides us with the average photon numbers of the output states, but not with their phases. Unlike in Fig. 8, we cannot use fidelity as a quality measure. Therefore, we will assess the performance of the RGQN using the following cost function:

6 $$\begin{aligned} \text {cost} = \sum _{k=0}^{K} |{\bar{n}}_\text {out}^k-{\bar{n}}_\text {target}^k| ~, \end{aligned}$$

where ${\bar{n}}_\text {out}^k$ and ${\bar{n}}_\text {target}^k$ are the average photon numbers of the *actual* output state and the *target* output state at iteration *k* respectively. *K* is the total number of states in the input time series. As the average photon number of a squeezed vacuum state increases monotonously with the squeezing parameter r^29^, i.e. ${\bar{n}} = \sinh ^2(r) > 0$, the cost function quantifies how much the squeezing levels of the output states deviate from the target values. As is explained in more detail in Methods, the experimentally obtained PNR results are re-scaled prior to evaluating Eq. (6) in order to compensate for hardware imperfections.

**Figure 10:**
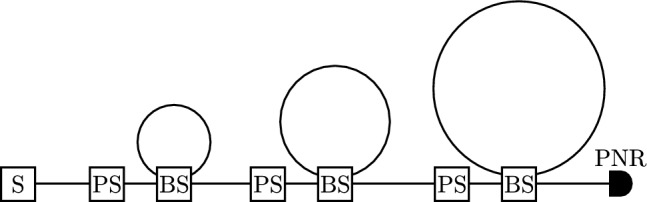
Borealis setup^15^. A squeezer (S) periodically produces single-mode squeezed states, resulting in a train of 216 states that are separated in time by $T = 36\mu s$. These states are sent through programmable phase shifters (PS) and beam splitters (BS). Each BS is connected to a delay line. From left to right in the figure, the lengths of these delay lines are *T*, 6*T*, and 36*T*. The output states are measured by a photon-number-resolving (PNR) detector.

The small-scale version of the QCE task (depicted in Fig. 9) is run on the photonic processor Borealis^15^ (depicted in Fig. 10). It consists of a single optical squeezer that generates squeezed vacuum states. These states are sent to a sequence of three dynamically programmable loop-based interferometers of which the delay lines have different lengths, corresponding with propagation times of *T*, 6*T*, and 36*T* ($T = 36 \mu s$). For our experiment, we only use the two leftmost loop-based interferometers. More formally, we choose $\theta =0$ for the rightmost BS and $\phi =0$ for the rightmost PS.

As is explained in more detail in Methods, we can virtually make the lengths of Borealis’ delay lines equal. We do so by lowering the frequency at which we send input states and by putting the $\theta =0$ for the leftmost beam splitter in between input times.

**Figure 11:**
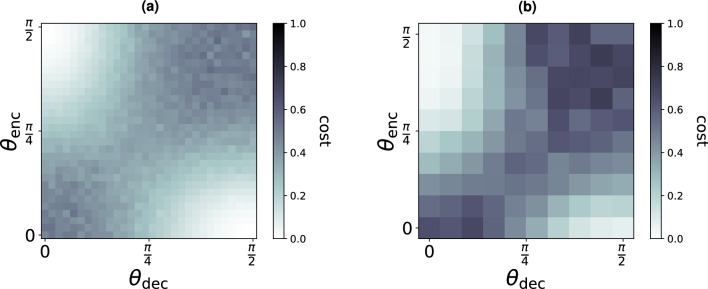
QCE performance of Borealis when $D=1$ and $\phi _{\text {enc}}= \phi _{\text {dec}}= 0$. (**a**) Shows the results from numerical simulations, while (**b**) Shows experimental results. The cost function is defined in Eq. (6). The optimal performance occurs when the input states are delayed by $D=1$ in the encoder and fully transmitted in the decoder ($\theta _{\text {enc}}=\pi /2$, $\theta _{\text {dec}}=0$), or when they are fully transmitted in the encoder and delayed by $D=1$ in the decoder ($\theta _{\text {enc}}=0$, $\theta _{\text {dec}}=\pi /2$).

We first consider the case where $\phi _{\text {enc}}= \phi _{\text {dec}}= 0$, such that all phase shifters can be disregarded. Figure 11 compares the experimental and numerical performance of the QCE task for $D=1$. We observe that the task is solved perfectly when either the encoder or the decoder delays states by $D=1$ and the other RGQN transmits states without delay. The performance is worst when both the encoder and decoder delay states with an equal number of iterations (either $D=0$ or $D=1$). Indeed, the cost of Eq. (6) is then evaluated between randomly squeezed vacuum states. For beam splitter angles between 0 and $\pi /2$, we find that the cost follows a hyperbolic surface. We observe good agreement between simulation and experiment.

We now consider the case where $\phi _{\text {enc}}$ and $\phi _{\text {dec}}$ are not restricted to 0. Note that the Borealis setup (Fig. 10) does not have phase shifters inside the loops. However, as is explained in more detail in Methods, we can *virtually* apply the phase shifts $\phi _{\text {enc}}$ and $\phi _{\text {dec}}$ *inside* Borealis’ loops by dynamically adjusting the phase shifters positioned *before* those loops over time. Unfortunately, this procedure is hampered by hardware restrictions. The range of Borealis’ phase shifters is restricted to $[-\pi /2,\pi /2]$. This is problematic, since in order to apply a single virtual phase shift value within a specific loop, the phase shifters preceding that loop need to be tuned dynamically across the complete $[-\pi ,\pi ]$ range. As a result, about half of the parameter values of the phase shifters cannot be applied correctly. When such a value falls outside of the allowed range, an additional phase shift of $\pi$ is added artificially to ensure proper hardware operation.

**Figure 12:**
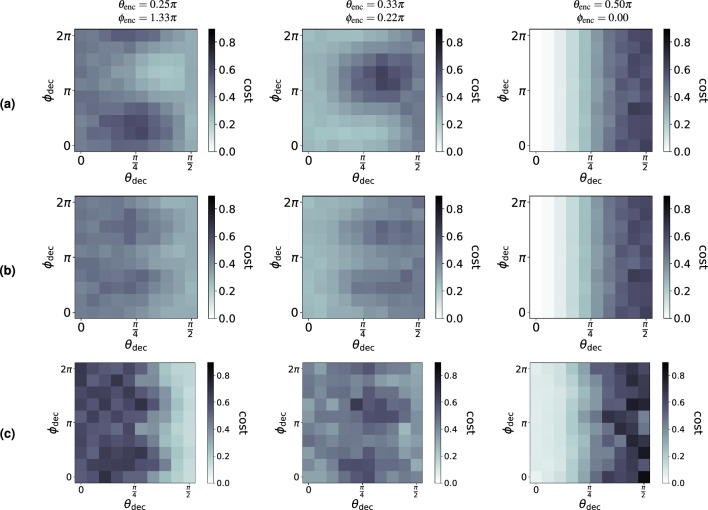
QCE performance of Borealis when $D=1$. (**a**) Simulation where phase shifters are tunable without range restriction. (**b**) Simulation where phase shifters are tunable within $[0,\pi )$. (**c**) Experiment where phase shifters are tunable within $[0,\pi )$. The columns correspond with different encoders. Each plot shows the QCE cost (as defined in Eq. (6)) as a function of the decoder parameters $\theta _{\textrm{dec}}$ and $\phi _{\textrm{dec}}$.

Figure 12 shows the QCE ($D=1$) performance for three different encoders (corresponding to the three columns). We compare classical simulation results (rows 1 and 2) with experimental results (row 3). Whereas the restricted range of Borealis’ phase shifters is taken into account in rows 2 and 3, it is not in row 1. While Fig. 11 (for $\phi _{\text {enc}}= \phi _{\text {dec}}= 0$) showed that the decoder can easily be optimized by considering only $\theta _{\text {dec}}= 0$ and $\theta _{\text {dec}}= \pi /2$, this optimization is less trivial when $\phi _{\text {enc}}\ne 0$ and $\phi _{\text {dec}}\ne 0$. We observe that the general trends of the cost function landscapes agree between rows 2 and 3, although row 3 is affected by experimental imperfections.

## Conclusions

We have introduced a new model to process time series of quantum states. Similar to an existing RC-inspired model, no detectors are present in our model, which allows the processing to be performed online. This leads to conceptually different tasks than the ones considered by temporal models with detectors. We first probed two key functionalities of our model: the linear memory capacity and the capability to entangle distinct states within a time series. As expected, these functionalities benefit from an RNN structure where all internal interactions can be trained (rather than fixing a subset of those interactions to random values). More interestingly, the RGQN showed the ability to tackle two *quantum communication* tasks, a domain where optics naturally is the leading hardware platform. First, we demonstrated that the RGQN can enhance the transmission rate of a quantum memory channel with Gauss-Markov noise by providing it with entangled input states. The enhancement showed to be near-optimal as compared to previous studies, while our generation scheme of the input states is simpler (e.g., it does not require Bell measurements). Moreover, our scheme is more versatile as it is known reconfigurable circuits can adapt to fabrication imperfections^18,19.^ Second, we showed that the RGQN can mitigate undesired memory effects in quantum channels. This task could previously only be solved when redundantly encoded input signals could be provided, which often is not the case in real-world scenarios. A small-scale experiment of this last task was performed on the photonic quantum processor Borealis^15^.

## Methods

The classical simulations of our RGQN were carried out using the open-source photonic optimization library MrMustard^30^. This allows us to perform gradient-based optimizations of symplectic circuits and orthogonal symplectic circuits.

### STQM

As is explained in Ref.^11^, a special purpose cost function *f* can be used to solve the STQM task. *f* can be defined as a function of the submatrices of the orthogonal symplectic matrix that is associated with the circuit ${\textbf{O}}$. With slight abuse of notation, we write the orthogonal symplectic *matrix* that is associated with the *circuit* ${\textbf{O}}$ as:

7 $$\begin{aligned} {\textbf{O}} = \left( \begin{array}{ll} {\textbf{A}} &{} {\textbf{B}} \\ {\textbf{C}} &{} {\textbf{D}} \end{array}\right) ~. \end{aligned}$$

In contrast to Eq. (1), the quadratures are ordered here as follows: $(q_1,p_1,q_2,p_2,q_3,p_3,...)$, where $q_i$ and $p_i$ are the quadratures of mode *i*.

When the delay is nonzero ($D>0$), Ref.^11^ shows that the goal of the STQM task can be redefined as the following system of equations:

8 $$\begin{aligned} {\left\{ \begin{array}{ll} {\textbf{D}} \approx {\textbf{0}}~, \\ \textbf{C A}^{D-1} {\textbf{B}} \approx {\textbf{I}}~, \\ \textbf{C A}^{t} {\textbf{B}} \approx {\textbf{0}}~,~ \forall \, t \ne D-1~. \end{array}\right. } \end{aligned}$$

Hence, we choose the following cost function:

9 $$\begin{aligned} \begin{aligned} f\left( {\textbf{U}}\right) =&\; 0.5\Vert {\textbf{D}}\Vert +5\left\| \textbf{C A}^{D-1} {\textbf{B}}-{\textbf{I}}\right\| \\ +&\; 0.5\sum _{\begin{array}{c} 0 \le t < T \\ t \ne D-1 \end{array}}{\Vert {\textbf{C}}{\textbf{A}}^t {\textbf{B}}\Vert }, \end{aligned} \end{aligned}$$

where $\Vert \cdot \Vert$ is the Frobenius norm. Note that the last sum in Eq. (9) was not used in Ref.^11^, as these terms appeared to worsen the results. However, we have found that their inclusion is beneficial in our work. A numerical parameter sweep showed that the factors 0.5, 5, and 0.5 for the terms in Eq. (9), together with a value of $T=10$ yield good results.

The learning rate is initialized at a value of 0.01 and decreased throughout the training procedure until the cost function converges. For each value of $(m_\text {io},m_\text {mem})$ in Fig. 3, the training is repeated for an ensemble of 100 initial conditions of the RGQN. After training, a test score is calculated as the average fidelity over a test set of 100 states. The fidelity is calculated according to Ref.^23^. Only the lowest test score in the ensemble of different initial conditions is kept, as this corresponds to a degree of freedom that is exploitable in practice.

In order to evaluate the test score, squeezed thermal states are used as input. In the case of a single mode, we first generate a thermal state with an expected number of photons ${\bar{n}}_\text {th}$. Afterwards, the state is squeezed according to a squeezing magnitude $r_\text {sq}$ and a squeezing phase $\phi _\text {sq}$. The relevant parameters of this state generation process are chosen uniformly within the following intervals: ${\bar{n}}_\text {th} \in [0,10)$, $r_\text {sq} \in [0,1)$ and $\phi _\text {sq} \in [0,2\pi )$.

If $m_\text {io}>1$, the state generation is altered as follows. We first generate a product state of single-mode thermal states. The *M*-mode product state is then transformed by a random orthogonal symplectic circuit, followed by single-mode squeezers on all modes. A second random orthogonal symplectic circuit transforms the multi-mode state to the final input state.

### Entangler task

We evaluate the cost function as follows. We send vacuum states to the RGQN for 10 iterations, such that the model undergoes a transient process. We numerically check that this transient process has ended by monitoring the convergence of the average photon number in the output mode. We then calculate the logarithmic negativity of the 2-mode state formed by the output at iteration 10 and $10+S$.

The logarithmic negativity of a 2-mode state is calculated from the determinants of the state’s covariance matrix (and its submatrices) as described in Ref.^12^. We want to emphasize that the definition of the logarithmic negativity in Eq. (76) of Ref.^12^ involves a logarithm, which has previously been implemented in literature both as a natural logarithm^31^ and as a binary logarithm^32^. Given that the analysis of the RC-inspired model in Ref.^11^ utilized a natural logarithm^26^, we adopt the same convention such that our results can be compared directly. Also note that we do not calculate the negativity from the symplectic eigenvalues of the covariance matrix (as is done for example in Ref.^32^), which is beneficial for numerical optimization.

The cost function is defined as the negative of the logarithmic negativity. The training is performed using 4000 updates of ${\textbf{S}}$ and with a learning rate of 0.01. For each value of $(S,m_\text {mem})$ in Fig. 4, the training is repeated for an ensemble of 100 initial conditions of ${\textbf{S}}$. Again, only the lowest score in the ensemble is kept.

### Superadditivity task

For more details on the calculation of the optimal transmission rate of the quantum channel with Gauss-Markov noise, both with and without entangled input states, we refer to Ref.^25^.

For the case of entangled input, the optimization problem defined by Eq. 9 of this Reference is solved under the restriction that the covariance matrix $\varvec{\gamma }_\text {in}$ is produced by the RGQN. $\varvec{\gamma }_\text {mod}$ is a general covariance matrix that takes into account the required input energy constraint (Eq. 13 in Ref.^25^).

The cost function is defined as the negative of the transmission rate. The training is performed using 1000 updates of ${\textbf{S}}$ and with a learning rate of 0.5.

### Quantum channel equalization

In contrast to the STQM task, here we use the negative of the fidelity (between the desired output and the actual output) as a cost function, both during training and testing. The fidelity is calculated according to Ref.^23^. The input states are single-mode squeezed thermal states where ${\bar{n}}_\text {th}$, $r_\text {sq}$, and $\phi _\text {sq}$ are chosen uniformly within the following ranges: ${\bar{n}}_\text {th} \in [0,10)$, $r_\text {sq} \in [0,1)$ and $\phi _\text {sq} \in [0,2\pi )$.

${\textbf{O}}_\text {enc}$ and ${\textbf{O}}_\text {dec}$ are initialized randomly. Every epoch, 30 squeezed thermal states are sent through the RGQNs. In order to account for the transient process at the start of each epoch, the fidelity is only averaged over the last 20 output states.

The training is performed using 2000 updates of ${\textbf{O}}_\text {dec}$ and with a learning rate of 0.05. In Fig. 8, the training is repeated for an ensemble of 20 initial conditions of ${\textbf{S}}$ for every value of $(D,m_\text {mem,dec})$ and for each encoder initialization. After training, a test score is calculated by simulating a transient process during 10 iterations and averaging the fidelity over 100 subsequent iterations. The lowest test score in each ensemble is kept.

### Experimental demonstration of quantum channel equalization

This section describes the technical details of both the simulations and the experiments that were presented in the context of the QCE experiment. We first describe how the RGQN can be mapped onto the Borealis setup. Afterwards, we describe the input state generation, the post-processing of the PNR results, and some other relevant parameters for the simulations and experiments.

#### **Mapping the RGQN model on Borealis**

We map our model on the Borealis setup of Fig. 10. We choose $\phi =0$ and $\theta =0$ for the rightmost phase shifter and beam splitter respectively, such that these components and their corresponding delay line can be disregarded. After applying these changes, it is clear that the Borealis setup differs from the setup in Fig. 9 in two ways. First, the remaining delay lines of the Borealis setup have different lengths, which is not the case in Fig. 9. Second, Borealis does not have phase modulators in the delay lines. We can circumvent both of these problems as follows.

First, we can virtually increase the length of the leftmost delay line (i.e. the encoder delay line) from *T* to 6*T* by performing two changes: (1) we lower the frequency at which we input states from $\frac{1}{T}$ to $\frac{1}{6T}$ and (2) we store the encoder memory state as long as we do not input a new state. More formally, we do the following. Before generating a new input state, we put the values of the beam splitter angles to $\theta _\text {enc}$ (for the encoder) and $\theta _\text {dec}$ (for the decoder). Once a state enters the encoder, it interacts with the memory state, leading to an output state and a new memory state for the encoder. The output state of the encoder is sent to the decoder. We now put $\theta _{\text {enc}}=0$ for a period of 6*T*, such that no new inputs can enter the loop. Hence, the new memory state is stored in the delay line of the encoder. During this period, no states are generated and no interactions occur between the encoder’s memory mode and input/output mode. Afterward, a new state is generated and the process is repeated.

Second, we can apply virtual phase shifts in the delay lines of Fig. 10 by using the phase shifters in front of the loops. To do this, we utilize an existing functionality of Borealis’ control software. This functionality (implemented in StrawberryFields^33^ as compilers.tdm.Borealis.update_params) is normally used to compensate for unwanted phase shifts in the delay lines. Given such a static unwanted phase shift, the compensation is performed by adjusting the phase shifters in front of the loops *over time*, such that they apply different phase shifts at different iterations. The actual unwanted phase shifts that are present in the hardware can be measured before running an experiment. We now choose to operate the setup *as if* there were phase offset sets $\phi _\text {1,unwanted} - \phi _\text {enc}$ and $\phi _\text {2,unwanted} - \phi _\text {dec}$ in the first two delay lines respectively. Hence, we apply net virtual phase shifts in the loop with values $\phi _\text {enc}$ and $\phi _\text {dec}$.

Unfortunately, this procedure is undercut by a hardware restriction of Borealis. The range of these phase shifters is limited to $[-\pi /2,\pi /2]$, which means that about half of the desired phase shifts cannot be implemented correctly. When a phase shift falls outside of the allowed range, an additional phase shift of $\pi$ is added artificially to ensure proper hardware operation. Listing 1 shows the Python code for this process.


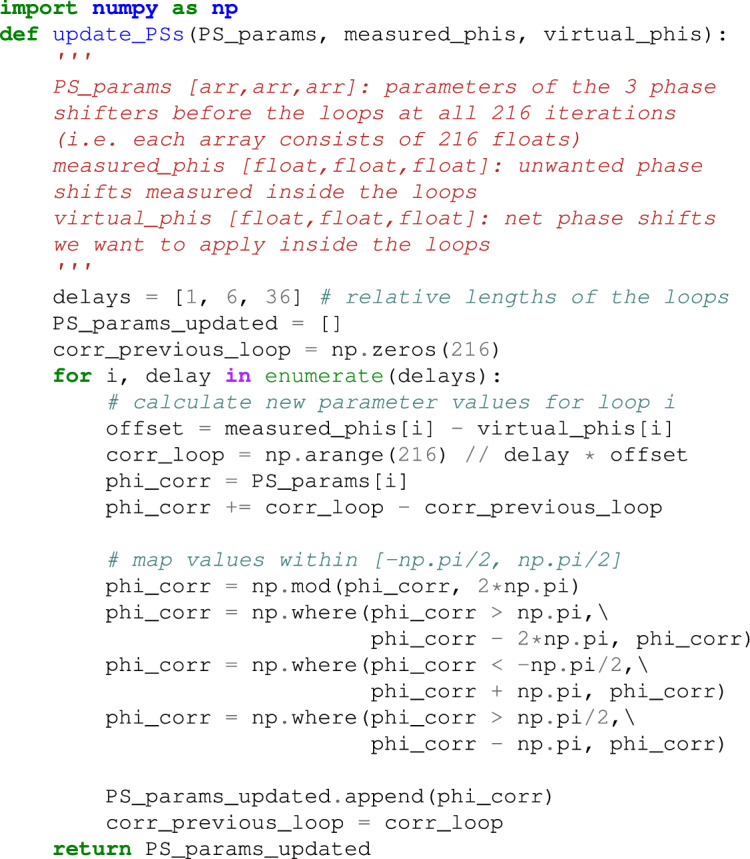

- Listing 1. Code to apply virtual phase shifts inside Borealis’ loops. This is an adapted version of the function compilers.tdm.Borealis.update_params of StrawberryFields^33^.

#### **Generating input states, post-processing PNR results, and simulation parameters**

Both for the computer simulations and for the experiments presented in the section on experimental QCE, the input states are generated by a single-mode optical squeezer that operates on a vacuum state. In every iteration, the squeezing magnitude is chosen randomly as either 0 or 1, effectively encoding bit strings into the quantum time series. It is worth noting that public online access to Borealis does not allow for multiple squeezing magnitudes within one experiment. Our experiments were therefore performed on-site.

The output of Borealis consists of the average photon numbers gathered over 216 iterations. Due to the mapping described in the previous section, only 36 of these average photon numbers can be used. To account for the transient process, the results of the first 10 of these 36 output states are disregarded. The PNR results of the remaining 26 output states are used to estimate the decoder’s QCE performance.

For the *simulations*, 10 runs (each using a different series of input states) are performed per set of gate parameter values of the RGQNs. The cost is therefore averaged over 260 output states. For the *experiments* shown in Figs. 11 and 12, the number of runs per data point is 7 and 2 respectively.

In hardware experiments, non-idealities occur that are not considered in the classical simulations. We compensate for two such non-idealities by re-scaling the experimentally obtained average photon numbers. On the one hand, there are optical losses. On the other hand, the optical squeezer suffers from hardware imperfections that lead to effective squeezing magnitudes that decrease over time when the pump power is kept constant. The re-scaling is performed as follows. At each iteration, we calculate the average of the number of detected photons over all experiments. We fit a polynomial of degree 2 to these averages. Given an experimental result, i.e. a series of average photon numbers, we then divide each value by the value of the polynomial at that iteration. The resulting series is rescaled such that the average of the entire series matches the average value of the entire series of input states.

## Data Availability

Data is available from the corresponding author upon reasonable request.
